# Increased Risk of Primary Sjögren's Syndrome in Female Patients with Thyroid Disorders: A Longitudinal Population-Based Study in Taiwan

**DOI:** 10.1371/journal.pone.0077210

**Published:** 2013-10-18

**Authors:** Ming-Chi Lu, Wen-Yao Yin, Tzung-Yi Tsai, Malcolm Koo, Ning-Sheng Lai

**Affiliations:** 1 Division of Allergy, Immunology and Rheumatology, Buddhist Dalin Tzu Chi Hospital, Chiayi, Taiwan; 2 School of Medicine, Tzu Chi University, Hualien, Taiwan; 3 Division of General Surgery, Buddhist Dalin Tzu Chi Hospital, Chiayi, Taiwan; 4 Department of Medical Research, Buddhist Dalin Tzu Chi Hospital, Chiayi, Taiwan; 5 Department of Nursing, Tzu Chi College of Technology, Hualien, Taiwan; 6 Department of Environmental and Occupational Health, College of Medicine, National Cheng Kung University, Tainan, Taiwan; 7 Dalla Lana School of Public Health, University of Toronto, Ontario, Canada; National Institute of Dental and Craniofacial Research, United States of America

## Abstract

**Background:**

A number of reports have indicated an association between thyroid diseases and primary Sjögren's syndrome (pSS). However, fewer studies have investigated whether the presence of thyroid diseases is associated with increased risk of developing pSS. Thus, the aim of our study was to use a nationwide health claims database to explore the prevalence and risk of pSS in female patients with thyroid diseases.

**Methods:**

From the Registry of Catastrophic Illness database in the National Health Insurance Research Database in Taiwan, we identified 389 female patients with a diagnosis of pSS from 2005 to 2010. We also obtained 1945 control subjects frequency-matched on sex, 10-year age interval, and year of index date from the Longitudinal Health Insurance Database (LHID2000). Both groups were retrospectively traced back to a period of eight years to obtain diagnosis of thyroid diseases prior to index date.

**Results:**

A significantly higher risk of pSS was associated with the presence of thyroid diseases (adjusted odds ratio (AOR) = 2.1, 95% confidence interval (CI) = 1.6–2.9). Among the sub-categories of thyroid diseases, patients with thyroiditis (AOR = 3.6, 95% CI = 1.7–7.5), thyrotoxicosis (AOR = 2.5, 95% CI = 1.6–3.8), and unspecified hypothyroidism (AOR = 2.4, 95% CI = 1.2–4.6), and simple and unspecified goiter (AOR = 2.0, 95% CI = 1.3–3.3) were significantly associated with increased risk of pSS. The associations were generally stronger in the mid-forties to mid-sixties age group, except in patients with unspecified hypothyroidism.

**Conclusions:**

The risk of pSS was significantly increased in female patients with thyroid diseases, particularly those in their mid-forties to mid-sixties. An increased awareness of the possibility of pSS in perimenopausal females with thyroid diseases is important to preserve their quality of life and to avoid comorbidity.

## Introduction

Sjogren's syndrome (SS) is a chronic inflammatory autoimmune exocrinopathic disease [1]. The disease can occur alone as primary SS (pSS) or in association with other autoimmune diseases as secondary SS. Depending on its diagnostic criteria and the population studied, SS was estimated to affect between 0.05% and 4.8% of adults [2]. Its prevalence increased with age and peak around age 55 to 64 years with a female to male ratio of 9.9 to 1 [3].

Although the disease affects primarily the salivary and lachrymal glands in its early stage, it can subsequently involve other organs or systems of the body such as the lungs, kidneys, thyroid, the circulatory system, and central nervous system [4]. Of these, the thyroid gland is of concern because of its histological and functional similarities with the lacrimal and salivary glands [5] and the coexistence of thyroiditis in patients with SS [6], and vice versa [7]. Furthermore, the histopathologic lesions of pSS and autoimmune thyroiditis showed infiltration of activated T lymphocytes suggesting that they could share pathogenic mechanisms and antigens [8], [9].

The prevalence of autoimmune thyroid disease (AITD), thyroid dysfunction, and the presence of thyroid antibodies have been well documented in patients with pSS [10]. Although the majority of studies have revealed an increased risk of thyroid disorders in patients with pSS, the temporal sequence of pSS and AITD was not entirely clear. Tektonidou et al. demonstrated that patients with AITD had higher presence of anti-nuclear antibody and risk for developing systemic autoimmune diseases such as primary SS and systemic lupus erythematosus [7]. In addition, the presence of main clinical features of SS such as xerostomia and keratoconjunctivitis sicca is high among AITD patients [11]. Therefore, the presence of AITD might also be a risk factor in the development of pSS.

To our knowledge, no studies have investigated the association between pSS and thyroid disorders in female patients prior to their pSS diagnosis using population-based data. Thus, the aim of our case-control study was to use a nationwide health claims database to explore the prevalence and risk of pSS in female patients with thyroid diseases.

## Materials and Methods

### Data source

The Taiwan National Health Insurance (NHI) scheme is a mandatory single-payer health insurance system implemented in 1995. We used the catastrophic illness file of the National Health Insurance Research Database (NHIRD) to identify patients with primary SS. In Taiwan, patients afflicted with one of the 31 categories of major illnesses or injuries can apply for a catastrophic illness certificate (CIC). Patients are exempted from co-payment if their applications are approved by the Bureau of NHI [12].

The study protocol was reviewed and approved by the Institutional Review Board of the Buddhist Dalin Tzu Chi Hospital, Taiwan (No. A10104020). Since the NHIRD files contain only de-identified secondary data, the review board waived the requirement for obtaining informed consent from the patients.

### Cohort of patients with pSS and control subjects

From the catastrophic illness file, patients with pSS were identified as cases in this study. Review of application of CIC by the Bureau of NHI id based on the criteria of the American-European Consensus Group for pSS [13]. We excluded patients under the age of 20 years and those with a CIC for autoimmune diseases other than SS. In addition, we included only females in the study because of the high female to male sex ratios of SS and thyroid disorders [3], [14]. The date of application of CIC in approved patients was considered as the index date in the analysis.

For each pSS case, five controls were randomly selected from the 2000 Longitudinal Health Insurance Database (LHID2000) frequency-matched by 10-year age interval, sex, and the year of the index date (index year). The index date for each of the control subjects was assigned by using the date of a randomly selected ambulatory visit for a given index year.

### Identification of thyroid disorders

Both the cases and controls were linked to the LHID2000 to obtain their ambulatory care visit data occurred from 1997 to 2010. In other words, up to a period of eight years in ambulatory care data were retrospectively tracked for each cases and controls. The *International Classification of Diseases, Ninth Revision, Clinical Modification* (ICD-9-CM) codes were used to define the thyroid diseases including the disorders of thyroid gland (240–242, 244–246), simple and unspecified goiter (240), nontoxic nodular goiter (241), thyrotoxicosis (242), unspecified hypothyroidism (244.9), thyroiditis (245), and other disorders of thyroid (246). Congenital hypothyroidism (243) was excluded from our study. To increase the validity of the diagnosis of thyroid diseases ascertained from administrative data, we included only patients who had two or more instances of the same ICD-9-CM codes in their medical claims prior to the index date.

### Statistical analysis

Summary statistics are expressed as frequency and percentage for categorical data or mean ± standard deviation (SD) and median (minimum, maximum) for continuous variables, as appropriate. Fisher's exact test and Mann-Whitney U-test were used to compare categorical and continuous variables between patients with pSS and control subjects, respectively.

The association between pSS and various thyroid disorders were estimated using both univariate logistic regression analysis and multivariate logistic regression analysis adjusting for the potential confounding effects of age at diagnosis of pSS and urbanization levels. Urbanization levels were estimated based on a combination of population density, percentage of residents with college level or higher education, percentage of residents 65 years and old, percentage of residents who were agriculture workers, and the number of physicians per 100000 people [15]. Crude and adjusted odds ratios (OR) are presented with 95% confidence intervals (95% CI). We conducted further analysis separately by age group (20–44, 45–64, and 65–89 years) at diagnosis of pSS and by duration of tracked period (0–2.9, 3.0–5.9, and 6.0–7.9 years). A value of p<0.05 was considered statistically significant in all calculations. All statistical analyses were conducted using IBM SPSS Statistics software package, version 21.0 (IBM Corp., Armonk, NY, USA).

## Results

A total of 343 female patients with pSS aged 20 years and above were identified and compared with 1735 control subjects frequency matched by 10-year age interval, sex, and the index year. Their mean age (±SD) was 53.9±13.7 years and the median was 54 years (minimum-maximum, 20–89 years). Over the eight year period, the overall prevalence of disorders of thyroid gland (ICD-9-CM codes 240–242, 244–246) was 20.7% in the cases and 10.8% in the controls (Table 1). Both the crude and adjusted ORs (2.2) indicated a significantly higher risk of pSS (p<0.001) in patients with disorders of thyroid gland. Among the six sub-categories of thyroid diseases, the prevalence of thyrotoxicosis (ICD-9-CM code 242) in patients with pSS was highest (9.9%), followed by simple and unspecified goiter (ICD-9-CM code 240) (7.3%) prior to the diagnosis of pSS. The prevalence figures of the remaining four conditions ranged from 3.5% to 4.1%. The risk of pSS in patients with thyroiditis (ICD-9-CM code 245) was 3.6 times higher than in individuals without thyroiditis (p<0.001). The risk of pSS was also significantly higher in patients with thyrotoxicosis (ICD-9-CM code 242) (adjusted OR = 2.45, p<0.001), unspecified hypothyroidism (ICD-9-CM code 244.9) (adjusted OR = 2.35, p = 0.012), and simple and unspecified goiter (ICD-9-CM code 240) (adjusted OR = 2.02, p = 0.004). On the other hand, nontoxic nodular goiter (ICD-9-CM code 241) and other disorders of thyroid (ICD-9-CM code 246) were not significantly associated with pSS.

**Table 1:** Univariate and multivariate logistic regression analysis of thyroid disorders in female patients with primary Sjögren's syndrome.

Thyroid disorder (ICD-9-CM code)	Case	Control	Univariate regression		Multivariate regression^1^	
	N = 343	N = 1735	Crude OR (95% CI)	p-value	Adjusted OR (95% CI)	p-value
**Disorders of thyroid gland (240–242, 244–246)**			2.15 (1.59–2.91)	<0.001	2.14 (1.57–2.90)	<0.001
Yes	71 (20.7)	188 (10.8)				
No	272 (79.3)	1547 (89.2)				
**Simple and unspecified goiter (240)**			2.05 (1.27–3.31)	0.003	2.02 (1.25–3.27)	0.004
Yes	25 (7.3)	64 (3.7)				
No	318 (92.7)	1671 (96.3)				
**Nontoxic nodular goiter (241)**			1.42 (0.76–2.64)	0.277	1.36 (0.73–2.56)	0.335
Yes	13 (3.8)	47 (2.7)				
No	330 (96.2)	1688 (97.3)				
**Thyrotoxicosis (242)**			2.47 (1.62–3.77)	<0.001	2.45 (1.60–3.76)	<0.001
Yes	34 (9.9)	74 (4.3)				
No	309 (90.1)	1661 (95.7)				
**Hypothyroidism, unspecified (244.9)**			2.40 (1.23–4.69)	0.010	2.35 (1.20–4.60)	0.012
Yes	13 (3.8)	28 (1.6)				
No	330 (96.2)	1707 (98.4)				
**Thyroiditis (245)**			3.66 (1.74–7.74)	0.001	3.56 (1.68–7.54)	0.001
Yes	12 (3.5)	17 (1.0)				
No	331 (96.5)	1718 (99.0)				
**Other disorders of thyroid (246)**			1.64 (0.89–3.02)	0.116	1.55 (0.82–2.92)	0.176
Yes	14 (4.1)	44 (2.5)				
No	329 (95.9)	1691 (97.5)				

ICD-9-CM: International Classification of Diseases, Ninth Revision, clinical modification; OR: odds ratio; CI: confidence interval.

1 Controlling for age at diagnosis of primary Sjögren's syndrome and urbanization levels.

Results of the analysis conducted with subjects' age divided into three groups indicated that the significant associations primarily occurred in the 45 to 64 years age group (Table 2). Only unspecified hypothyroidism in the 20–44 years age group showed a significant association with pSS (p = 0.025) but the 95% CI was relatively wide (1.30–48.14). Neither the overall disorders of thyroid gland nor the six sub-categories of the thyroid disorders were significantly associated with pSS in the 65–89 years age group.

**Table 2:** Univariate logistic regression analysis of thyroid disorders in female patients with primary Sjögren's syndrome by age group.

Thyroid disorder (ICD-9-CM code)	Age (years)					
	20–44		45–64		65–89	
	(n = 520)		(n = 1087)		(n = 471)	
	OR (95% CI)	p-value	OR (95% CI)	p-value	OR (95% CI)	p-value
Disorders of thyroid gland (240–242, 244–246)	1.85 (0.96–3.56)	0.067	2.44 (1.66–3.60)	<0.001	1.64 (0.77–3.48)	0.199
Simple and unspecified goiter (240)	1.22 (0.40–3.70)	0.733	2.79 (1.56–4.99)	0.001	0.96 (0.21–4.42)	0.957
Nontoxic nodular goiter (241)	2.07 (0.40–10.86)	0.389	1.58 (0.76–3.26)	0.222	0.52 (0.06–4.14)	0.538
Thyrotoxicosis (242)	2.05 (0.88–4.79)	0.098	2.67 (1.56–4.58)	<0.001	2.42 (0.73–8.08)	0.150
Hypothyroidism, unspecified (244.9)	7.92 (1.30–48.14)	0.025	2.70 (1.18–6.15)	0.018	0.58 (0.07–4.66)	0.609
Thyroiditis (245)	2.60 (0.47–14.41)	0.275	4.62 (1.85–11.55)	0.001	1.77 (0.18–17.25)	0.623
Other disorders of thyroid (246)	1.47 (0.30–7.22)	0.633	1.46 (0.68–3.12)	0.332	2.71 (0.66–11.08)	0.166

To assess if the associations between the thyroid disorders and pSS varied with different durations prior to diagnosis of pSS, additional logistic regression analyses were conducted with tracking duration divided into three durations (Table 3). Results revealed that the associations were generally more apparent in the period closest to the diagnosis of pSS. The association between disorders of thyroid gland and pSS was the strongest in the 0–2.9 year period (OR = 2.32) compared with earlier periods. Among the sub-categories of thyroid disorders, simple and unspecified goiter showed similar pattern. Similarity, thyrotoxicosis and unspecified hypothyroidism were significantly associated with pSS only in the 0–2.9 year period. Thyroiditis was associated with pSS in both the 0–2.9 year (OR = 4.4, p = 0.009) and 6.0–7.9 year (OR = 3.1, p = 0.046) periods but not in the period (OR = 1.7, p = 0.531) in-between the two.

**Table 3:** Univariate logistic regression analysis of thyroid disorders in female patients by duration prior to diagnosis of primary Sjögren's syndrome.

Thyroid disorder (ICD-9-CM code)	Duration prior to diagnosis of primary Sjögren's syndrome (year)					
	0–2.9		3.0–5.9		6.0–7.9	
	OR (95% CI)	p-value	OR (95% CI)	p-value	OR (95% CI)	p-value
Disorders of thyroid gland (240–242, 244–246)	2.32 (1.65–3.26)	<0.001	1.84 (1.24–2.76)	0.003	1.46 (0.88–2.44)	0.143
Simple and unspecified goiter (240)	2.45 (1.38–4.33)	0.002	2.18 (1.10–4.33)	0.026	0.28 (0.04–2.06)	0.208
Nontoxic nodular goiter (241)	1.35 (0.61–2.96)	0.460	1.59 (0.63–4.00)	0.327	0.99 (0.29–3.45)	0.991
Thyrotoxicosis (242)	2.20 (1.29–3.76)	0.004	1.61 (0.88–2.97)	0.125	1.81 (0.84–3.90)	0.132
Hypothyroidism, unspecified (244.9)	2.29 (1.04–5.07)	0.041	1.43 (0.47–4.38)	0.528	2.86 (0.83–9.81)	0.095
Thyroiditis (245)	4.36 (1.46–13.03)	0.009	1.67 (0.34–8.31)	0.531	3.13 (1.02–9.62)	0.046
Other disorders of thyroid (246)	1.83 (0.85–3.95)	0.125	1.96 (0.82–4.73)	0.133	NA	NA

## Discussion

In this population-based sample of patients in Taiwan, we assessed the risk of pSS in patients with various thyroid disorders compared with patients without pSS. Overall, we found the disorders of thyroid gland (ICD-9-CM codes 240–242, 244–246) were significantly associated with pSS with an adjusted OR of 2.14. Specifically, thyroiditis (ICD-9-CM code 245), thyrotoxicosis (ICD-9-CM code 242), unspecified hypothyroidism (ICD-9-CM code 244.9), and simple and unspecified goiter (ICD-9-CM code 240) were significantly associated with pSS.

The increased risk of pSS in both overall disorders of thyroid gland and specific forms of thyroid disorders were likely to be related to AITD. Several studies have reported an increase in the risk of pSS in patients with AITD. In a study on 19 patients with autoimmune thyroiditis, six of them fulfilled the criteria of pSS [16]. In a case-control study, 40 women with five years history of postpartum thyroiditis were found to have significantly more impaired tear or saliva production and histological findings of SS compared with the controls [17]. Another study in Spain on 176 patients diagnosed with AITD, features of SS were diagnosed in 24% of the patients [11].

In our study, we observed that pSS was significantly associated with thyrotoxicosis (ICD-9-CM code 242) and simple and unspecified goiter (ICD-9-CM code 240). It is likely that these conditions were the results of Graves' disease [18]. Although it was not possible to identify the diagnosis of Graves' disease directly from the ICD-9-CM codes, it might be deduced based on the following reasons. Graves' disease is the most common cause of hyperthyroidism and the latter can lead to thyrotoxicosis. Graves' disease was also characterized by diffuse goiter. Nevertheless, we could not completely exclude the possibility of toxic thyroid nodule (also known as Plummer's disease) as a cause of thyrotoxicosis. Furthermore, the observation in our study that nontoxic nodular goiter, which is the enlargement of the thyroid gland not associated with abnormal thyroid function, did not increase the risk of pSS further supported that the role of AITD in pSS. Although the most common cause of endemic colloid goiter worldwide is iodine deficiency [19], goiter-related iodine insufficiency was rare in Taiwan because salt iodization has been widely implemented in Taiwan beginning in the mid-1960s.

Thyroiditis (ICD-9-CM code 245) and unspecified hypothyroidism (ICD-9-CM code 244.9) was found to be significantly associated with pSS in our study. Based on our clinical experience, acute or sub-acute thyroiditis was very rare and unlikely to be a cause of hypothyroidism. Again, the presence of iodine deficiency was unlikely in our study population. Therefore, the most common cause of the observed hypothyroidism is probably due to Hashimoto's thyroiditis. It should be noted that Hashimoto's thyroiditis was included under ICD-9-CM code of thyroditis (245) as 245.2. Therefore, it could be deduced that the underlying cause of the observed thyroiditis and unspecified hypothyroidism was likely to be due to Hashimoto's thyroiditis.

There is a body of literature indicating the association of Hashimoto's thyroiditis and systemic autoimmune diseases. Hashimoto's thyroiditis is an autoimmune disorder in which the lymphocytic infiltration of the thyroid gland leads to apoptosis of thyroid cells. Hashimoto's thyroiditis is also a cause of non-endemic goiter. In a prospective study of 1517 patients with systemic autoimmune diseases, the prevalence of Hashimoto's thyroiditis was found to be 176-fold higher in patients with SS than in the general population [20]. A cohort study of 131 female Taiwanese patients with pSS, rheumatoid arthritis, or systemic lupus erythematosus also revealed that the incidence of Hashimoto's thyroiditis was significantly higher in patients with pSS (20%) compared with the other two disorders (≤5%) [21]. Furthermore, a case-control study based on the analysis of the Taiwan's nationwide health claims database revealed that the prevalence of hypothyroidism in patients with pSS was 6.7% and was significantly higher than the 3.0% found in controls (p<0.001). The risk of hypothyroidism in patients with pSS, adjusted for sex, age, monthly income, and level of urbanization, was significantly higher compared to patients without pSS (OR = 2.37, 95% CI 1.92–2.93) [4]. Punzi et al. compared prevalence of thyroid disorders among 121 patients with pSS, 74 patients with rheumatoid arthritis, and 404 control subjects. Although both groups of patients had significantly higher frequency of antithyroglobulin antibodies, hypothyroidism was significantly more common among patients with pSS than those with rheumatoid arthritis [22].

Our finding that significant associations occurred predominantly in the 45–64 years age group at the time of diagnosis of pSS was consistent with the epidemiological features of pSS and thyroid diseases. The incidence of pSS increased with age and reached its peak at 55–64 years in women [3]. Similarly, the peak ages for diagnosis of Graves' disease and Hashimoto's thyroiditis were the fourth through sixth decades of life [23].

Furthermore, we found that the associations between pSS and thyroid diseases were generally more apparent in the period closest to the diagnosis of pSS. The finding suggested a time lag of around six years existed between pSS and thyroid disorders. Nevertheless, the progression of the clinical course of pSS is typically slow and the nature of its early manifestations might be minor and nonspecific. Consequently, delays of 5–10 years after symptom appearance prior to diagnosis of pSS are not uncommon [24]. On the other hands, the symptoms and signs of AITD such as hypothyroidism or hyperthyroidism could cause patients to seek for medical care. Therefore, our finding could not rule out the possibility that pSS and AITD could be occurring concurrently.

Several limitations should be taken into account when interpreting the results from this study. First, patients with minor manifestations of pSS might not have applied for a catastrophic illness certificate and therefore, could not be identified through the use of registry for catastrophic illness patients. Second, ICD-9-CM codes do not differentiate whether the forms of thyroid disorders are of AITD or non-AITD in nature. Third, clinical parameters or lifestyle variables such as smoking, alcohol use, dietary habits, physical activity, and family history were not available in the database.

In conclusion, the current study was, to our knowledge, the first to assess the association between various thyroid disorders in female patients with pSS using a population-based study. Our main finding was a significant increased risk of pSS in female patients with thyroid diseases, particularly those in mid-forties to mid-sixties. Although pSS is a relatively benign and non-life-threatening disease in itself, it can lead to complications such as keratoconjunctivitis, xerostomia, dental caries, and even an increased risk of lymphoma. Therefore, to improve quality of life and avoid complications, an increased awareness of the possibility of pSS in perimenopausal females with thyroid diseases is important.
